# Post-cataract endophthalmitis caused by multidrug-resistant *Stenotrophomonas maltophilia*: clinical features and risk factors

**DOI:** 10.1186/1471-2415-15-14

**Published:** 2015-01-24

**Authors:** Yinghong Ji, Chunhui Jiang, Jian Ji, Yi Luo, Yongxiang Jiang, Yi Lu

**Affiliations:** Department of Ophthalmology, Eye & ENT Hospital, Fudan University, No. 83 Fenyang Road, Shanghai, 200031 China; Clinical Laboratory, Eye & ENT Hospital, Fudan University, No. 83 Fenyang Road, Shanghai, 200031 China

**Keywords:** Cataract surgery, Endophthalmitis, Intraocular lens, Drug resistance, *Stenotrophomonas maltophilia*

## Abstract

**Background:**

To report clinical features and risk factors of post-cataract surgery endophthalmitis (PE) due to *Stenotrophomonas maltophilia*.

**Methods:**

A retrospective case review from December 10, 2010 to April 7, 2011 was performed at the Eye & ENT Hospital, Fudan University. Data were collected for surgical details, disease characteristics, antibiotic sensitivity of the pathogen, and treatment response. Visual outcomes were examined with a minimum follow-up of 12 months.

**Results:**

Fourteen cases of *S. maltophilia* endophthalmitis were identified. The onset of infection occurred from 1–56 days postoperatively (median, 13.5 days). Obvious cellular reactions were found in all patients in the anterior chamber, along with the absence of pupil synechia. Retinal periphlebitis was an early sign of PE. *S. maltophilia* was positive in eight patients (57.1%). The fluids from aspiration tubes revealed the same bacteria, which were resistant to multiple drugs (e.g., amino glycosides, most of the β-lactams, aztreonam, imipenem, and ciprofloxacin), except levofloxacin. Compared with the culture-negative group, the infection was more rapid, more severe, and more difficult to control in the culture-positive group. Among 14 patients, 11 patients (78.6%) underwent pars plana vitrectomy (PPV) with intravitreal injection. Three patients had PPV twice, and three patients had intraocular lens and capsular bag removal. A final visual acuity of ≥20/100 was achieved by 13/14 patients (92.9%). Complications included retinal detachment in three cases (21.4%) and recurrence of infection in two cases (14.8%). Statistical analysis showed that age over 90 years and posterior capsule rupture were risk factors of infection (P *=* 0.034 and P *=* 0.034, respectively). The phacoemulsifier allowed potential contamination between the aspiration and irrigation tubes.

**Conclusions:**

*S. maltophilia* should be considered a pathogenic organism of PE. The infection often occurs in older patients with posterior capsule rupture. Intravitreal or systemic administration of effective antibiotics and earlier initial PPV may contribute to better clinical outcomes. Tubes with connections between aspiration and irrigation should be avoided during surgery.

## Background

Post-cataract surgery endophthalmitis (PE) is a serious complication with guarded prognosis for useful visual acuity. The reported incidence rates after cataract surgery vary between countries, and range from 0.28 − 8 per 1000 individuals [1–3] and 0.33 per 1000 individuals from 2006 to 2011 in China [4]. The microorganisms involved in this disorder are usually the endogenous colonies that exist in the patients’ eyelids and conjunctiva. However, they could also have originated from unusual exogenous sources, e.g., contaminated surgical materials, ocular rinsing solutions, or a contaminated environment. *Staphylococcus epidermidis* is the most common pathogen isolated in culture-proven endophthalmitis (60% of cases), with *Staphylococcus aureus*, *Streptococcus spp.*, and *Enterococcus spp*. each found in 5%–10% of the cases. Other Gram-negative bacteria account for approximately 6% of the cases [5].

*Stenotrophomonas maltophilia* (*S. maltophilia*) is an aerobic, nonfermentative, Gram-negative bacterium. Initially classified as *Pseudomonas maltophilia*, it was once grouped in the genus *Xanthomonas* [6]. It is ubiquitous in aqueous environments, soil, and plants. *S. maltophilia* has gained importance as a hospital pathogen because of its ability to colonize on plastic, glass, and Teflon [7]. It can cause ocular infections such as keratitis, conjunctivitis, and scleritis. There have been a few sporadic cases of iatrogenic *S. maltophilia*-induced PE since its first description in 1997 [8–12]. Only two studies involved an outbreak in more than three patients [13, 14], and only one study found the source of the infection, which was the rinsing solution [14]. Currently, there have been no reports describing the symptoms of this bacterial infection. The purpose of this study was therefore to summarize the clinical features of PE caused by *S. maltophilia*, and to investigate the risk factors of the outbreak. In addition, the potential source of contamination was also identified.

## Methods

### Patients

The patients were diagnosed and treated for endophthalmitis after receiving cataract surgery at the Eye & ENT Hospital, Fudan University, Shanghai, China, between December 10, 2010 and April 7, 2011. This study was designed to collect clinical data at the time of admission and during a minimum follow-up of 12 months. Patients without vitreous inflammation were excluded. Data collected included demographic information, eyes affected, date of surgery, surgical complications, type of intraocular lens implanted, operating room (OR) number and phaco machine, time between cataract surgery and diagnosis of endophthalmitis, site of culture, antibiotic sensitivity testing, treatment, and outcomes. All patients signed the informed consent form. The study protocol followed the tenets of the Declaration of Helsinki and was approved by the ethics committee of Eye & ENT Hospital, Fudan University (IRB number: KJ2010-41).

### Preoperative details

Preoperatively, 0.3% ofloxacin eye drops were used three times a day for 3 days. Intraoperatively, povidone-iodine solutions were applied three times to conjunctiva, lids, eyelashes, and periorbital areas. Norvancomycin (4 mg) was used in the irrigation fluid (500 ml) to prevent infection by Gram-positive organisms. All surgical instruments and phaco tubes were sterilized using full-cycle steam sterilization. A new drainage bag or cassette of the phaco machine was used for multiple patients on the same day, but was discarded at the end of the day. All other instruments [e.g., syringes, viscoelastic agents, and IOL (intraocular lens) cartridges] were disposable. At the conclusion of surgery, Tobradex ointment was applied. Postoperatively, Tobradex and Pranoprofen eye drops were used to decrease inflammation.

### Microbiological analysis

After noting endophthalmitis, sterilization procedures in the hospital were reviewed. To determine the origin of the *S. maltophilia*, multiple surveillance samples were collected from the air, from disinfectants, from the hands of surgeons, from the povidone-iodine solutions, from irrigation solutions, from viscoelastic agents, and from tubes and various instruments. At the beginning, only the irrigation tubes and other surveillance samples were tested and the results were negative. The fluids collected from aspiration tubes of phacoemulsifiers were not analyzed until a specialist in hospital hygiene was consulted on April 1, 2011. On April 7, a report showed that multidrug-resistant *S. maltophilia* was positive from the aspiration tube of one phacoemulsifier (Stellaris; Bausch & Lomb, Rochester, NY, USA). The planned surgeries using that machine were cancelled immediately on April 8, with no subsequent occurrence of endophthalmitis. Vitreous and aqueous fluids from tap or pars plana vitrectomy (PPV) were collected in the operating room using a sterile syringe before the administration of intravitreal antibiotics. Air contamination was measured with agar plates by the sedimentation method, using a 5-minute exposure of plates. Concentrations of airborne bacteria and fungi were expressed as colony-forming units per cubic meter (CFU/m^3^) by the formula CFU/m^3^ = 50000 N/(A*T), where N was the average CFU of plates, A was the plate area (cm^2^), and T was the exposure time (minutes). The collected fluids from different sources were inoculated on Columbia blood agar with 5% sheep erythrocytes (BioMerieux, Marcy l'Etoile, France), at 37°C, overnight. MicroScan autoScan-4 (Siemens Healthcare Diagnostics, Deerfield, IL, USA) was used for identification of all positive cultures. In accordance with the guidelines of the Clinical and Laboratory Standards Institute (CSLI), antibiotic susceptibility was tested by a broth dilution susceptibility test with NC 31 (Siemens Healthcare Diagnostics). The culture media were incubated for 7 days, and when negative was reported as “no growth”.

### Treatments

The initial antibiotic treatment was topical, using fortified levofloxacin (5 mg/ml), tobramycin (3 mg/ml), intravenous ceftriaxone or ceftazidime (40 mg/kg), and intravitreal antibiotics (Norvancomycin 0.8 mg/0.1 ml and ceftazidime 2.25 mg/0.1 ml). Later, the antibiotics were changed on the basis of the antibiotic susceptibility test. PPV or IOL extraction was performed when the clinical course of infection led to a suspicion of impending blindness. Immediate PPV was first performed in patients who had light perception (LP) visual acuity, then later in patients with the visual acuity of hand motion (HM). IOLs were extracted from a diabetic patient with severe inflammation and a highly myopic patient with a negative power of the IOL.

### Statistics

Statistical analysis included Fisher’s exact test and the Mann–Whitney *U* test. They were two-tailed, and significance was defined as P values less than 0.05. Statistical analysis was performed using the SPSS program for Windows (SPSS Institute, Chicago, IL, USA).

## Results

### Baseline data

The incidence of endophthalmitis was 14/468 (2.99%) for the machine during the 4-month period. For the 14 patients in this study (five women and nine men), operations were performed by five different surgeons, in two different operating rooms. All injected and foldable IOLs were from six different companies. The average age of patients was 64.6 years (range 39–93 years). Two patients were over 90 years of age. The IOL was implanted in the sulcus in two eyes with posterior capsular rupture due to eye trauma or cataract surgery. Another 12 patients had uneventful surgeries. Five patients had high myopia. The period from surgery to presentation was 1–56 days (median 13.5 days). Thirteen patients were acute onset (<6 weeks), and only one patient was delayed onset (>6 weeks). All patients initially reported blurred vision, and the presenting visual acuity (VA) was less than or equal to hand motion (HM) in nine of 14 patients (64.3%, Table 1).

**Table 1:** **Demographics and clinical data of 14 patients with post-cataract surgery endophthalmitis caused by** ***S. maltophilia***

Pt. no.	Ocular history	PCR	Days to infection	Initial VA	Final VA
1	-	-	10	HM	20/60
2	-	-	13	HM	20/100
3	High myopia	+	13	HM	20/100
4	High myopia	-	3	HM	20/60
5	PDR	-	1	HM	HM
6	High myopia	-	9	HM	20/60
7	-	-	9	20/125	20/25
8	High myopia	-	17	20/60	20/25
9	Ocular trauma	+	16	HM	20/60
10	High myopia	-	14	20/200	20/40
11	-	-	18	20/40	20/20
12	-	-	22	LP	20/50
13	-	-	28	HM	20/100
14	-	-	56	20/250	20/40

Pt. = patient; no. = number; PCR = Posterior capsule rupture; PDR = proliferative diabetic retinopathy; HM = hand motion; LP = light perception; VA = visual acuity.

### Clinical symptoms

**Figure 1:**
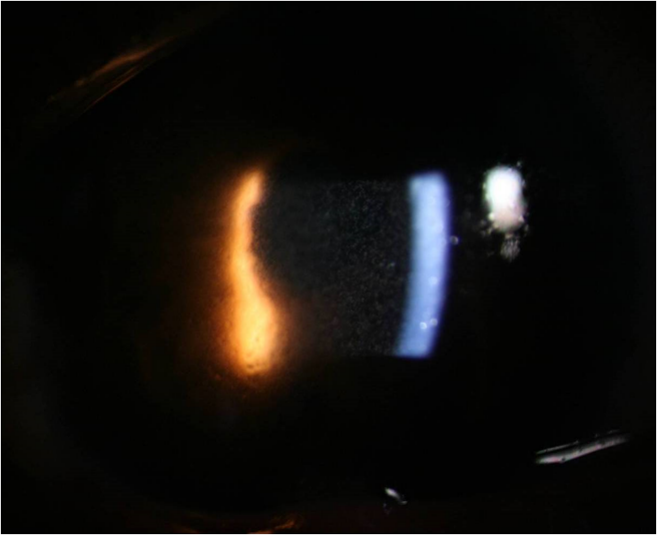
**Slit lamp photograph of patient 1.** Cells were found in the anterior chamber.

**Figure 2:**
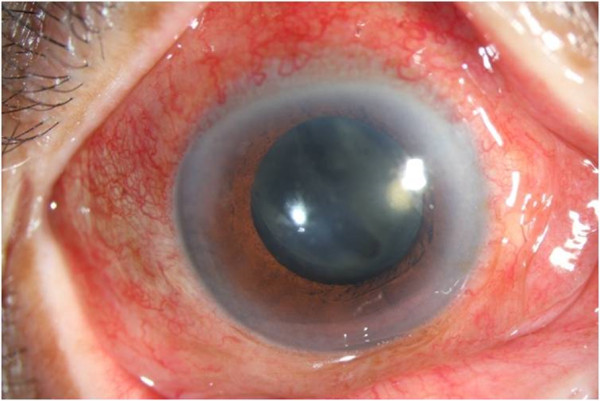
**A thin fibrin membrane was deposited on the surface of the intraocular lens.**

**Figure 3:**
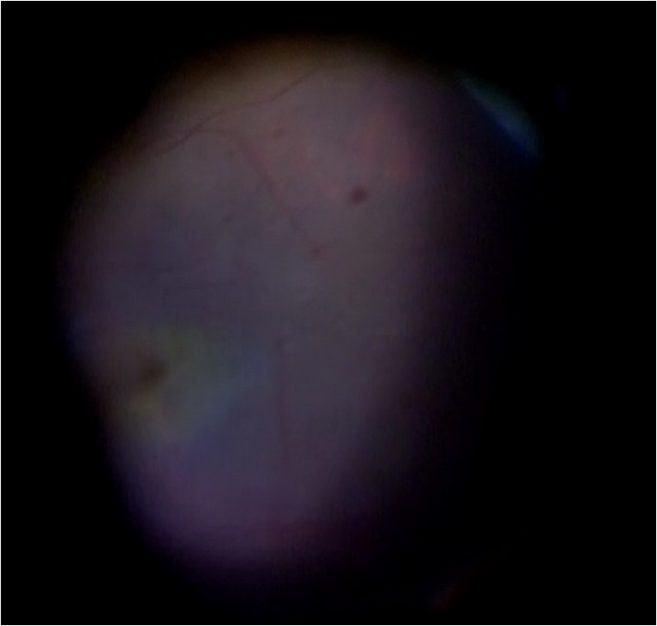
**Retinal periphlebitis was observed during vitrectomy surgery.**

Fourteen patients developed clinically diagnosed endophthalmitis, which included pain, decreased visual acuity, diffuse bulbar conjunctival hyperemia or chemosis, inflammation of the anterior segment (anterior chamber reaction including one of the following: flare, cells, hypopyon, fibrin, or pupillary fibrin membrane), and posterior segment inflammation (all patients had vitreous infiltration diagnosed by biomicroscopy or by ophthalmic ultrasound). Corneal edema occurred in four patients (28.6%). No patients had corneal infiltrates or cataract wound abnormalities. Initially, all patients had marked anterior chamber inflammation, with cells more than 2+ (Figure 1). Hypopyon was seen in nine patients (64.3%), with a height of approximately 2 mm. Four patients (28.6%) developed thin fibrin formation (Figure 2). Pupil synechia was not seen in any patient. Within 48 hours, there was vitreous inflammation and loss of posterior view, and vitritis varied in different patients. All patients had retinal periphlebitis, which was found in three cases without PPV preoperatively and 11 cases with PPV during surgery (Figure 3). The extent of retinal periphlebitis was not proportional to the vitreous inflammation.

### Treatment course and visual prognosis

The results of culture for the 14 patients could be classified into two groups. The culture positive group was composed of eight patients positive for *S. maltophilia,* four from tap and the other four from PPV. The culture-negative group was composed of six patients, three from tap and the other three from PPV. There was no significant difference between the two groups regarding the source of culture (P *=* 1.000, Fisher’s exact test). The interval between surgery and the beginning of symptoms was shorter for the culture-positive group (1–17 days, median 9.5 days), compared with 14–56 days (median 20 days) for the culture-negative group (P *=* 0.004, Mann–Whitney *U* test) (Table 2). All strains of *S. maltophilia* were sensitive to levofloxacin, two cases were sensitive to ceftazidime, and one case was sensitive to sulfamethoxazole (SMZ)/trimethoprim (TMP) (Table 3). The microbial susceptibility testing of *S. maltophilia* resulted in a change of therapy to systemic and intravitreal levofloxacin. Based upon past studies, a concentration of 0.5 mg/0.1 ml of intravitreal levofloxacin was used [15, 16], resulting in a decrease in the infection with no evidence of retinal toxicity.

**Table 2:** **Differences in patients from culture-positive and -negative groups**

Group	No. patients	***S. maltophilia***	Median latent days	PPV required	Two or more surgeries	IOL extraction	Recurrence
A	8	*+*	9.5 d	8	6	3	2
B	6	*_*	20 d	3	0	0	0

No. = number; PPV = pars plana vitrectomy; IOL = intraocular lens.

**Table 3:** **Antibiotic susceptibility testing of** ***S. maltophilia*** **isolates from eight eyes and from the aspiration tube**

Antibiotic drug	Pt. 1	Pt. 2	Pt. 3	Pt. 4	Pt. 5	Pt. 6	Pt. 7	Pt. 8	Aspiration
	(V)	(V)	(A)	(V)	(V)	(V)	(V)	(V)	
Gentamicin	R	R	R	R	R	R	R	R	R
Tobramycin	R	R	R	R	R	R	R	R	R
Amikacin	R	R	R	R	R	R	R	R	R
Ceftriaxone	R	R	R	R	R	R	R	R	R
Ceftazidime	I	R	S	R	R	R	R	S	R
Imipenem	R	R	R	R	R	R	R	R	R
Ciprofloxacin	R	R	R	R	R	R	R	R	R
Levofloxacin	S	S	S	S	S	S	S	S	S
SMZ/TMP	R	R	S	R	R	R	R	R	R

Pt. = patient; V = vitreous sample; A = aqueous humor sample; SMZ/TMP = sulfamethoxazole/ trimethoprim; S = sensitive; I = intermediate sensitivity; R = resistant.

In the culture-positive group, patients 1, 2, 7, and 8 initially received intravitreal injections without PPV. However, the infection worsened or did not improve, requiring PPV with additional intravitreal injections. Patient 2 had a recurrence of infection, and a second PPV with another intravitreal injection was performed. The other four patients (patients 3–6) started with intravitreal injections and PPV simultaneously. Patient 3 had a posterior capsular rupture during cataract surgery, and his infection was refractory. After two PPVs with intravitreal injections within 17 days, the infection still recurred, and did not resolve until the IOL and capsular bag were extracted. Patient 4, with a negative power of IOL, and patient 5, with diabetes, were more severe, and they also underwent IOL and capsular extraction. The infection of patient 5 was difficult to control and required more intravitreal injections after PPV. Later the patient had total tractional retinal detachment, and underwent another PPV with silicone oil tamponade. In the culture-negative group, six patients were treated once with initial intravitreal injections or PPV, which controlled the infection. The infection was therefore easier to control in the culture-negative group than in the culture-positive group (P *=* 0.016, Mann–Whitney *U* test).

The final VA varied from HM to 20/20. Five patients (35.7%) had a VA equal to or better than 20/40, and 13 patients (92.9%) had a VA equal to or better than 20/100 (Table 1). The two groups had similar visual prognoses (P *=* 0.264, Mann–Whitney *U* test). Two of the fourteen patients (14.3%) suffered from rhegmatogenous retinal detachment at 42 and 34 days. Both patients underwent another PPV with silicone oil or gas tamponade.

### Risk factors and the source of infection

**Figure 4:**
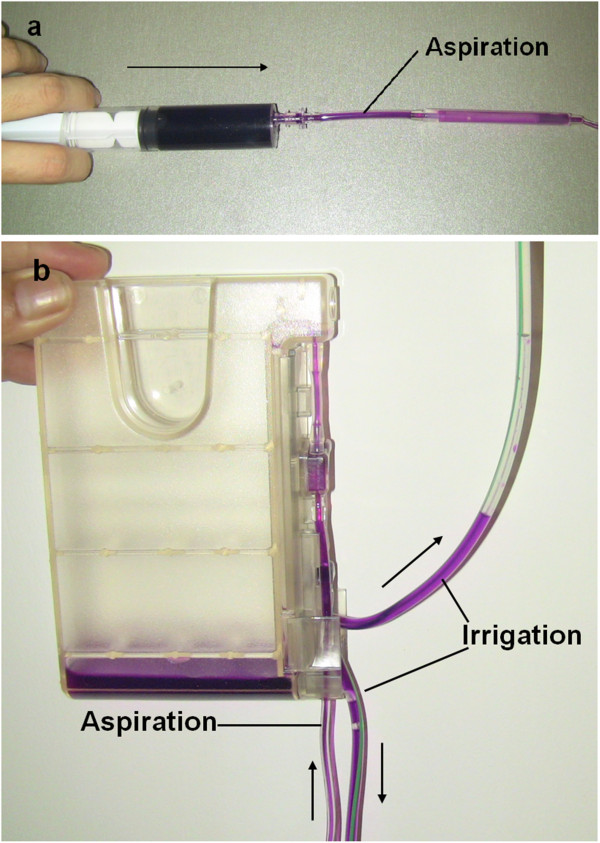
**The connection between the Aspiration tube and Irrigation tube. (a)** Fluid containing dye was injected into the aspiration tube with a syringe. **(b)** The fluid containing dye can be seen traveling from the aspiration tube to the irrigation tube of the drainage cassette assembly, with an I/A set (BL5113) from a Stellaris 1.8-mm Stablechamber phaco pack with a MICS needle. The arrow shows the direction of the fluids.

There was no correlation between high myopia and endophthalmitis (P *=* 0.538, Fisher’s exact test). Older age (i.e., over the age of 90 years) and posterior capsule rupture were risk factors for infection (P *=* 0.034 and P *=* 0.034, respectively, Fisher’s exact test). All patients were treated with the same phacoemulsifier (Stellaris; Bausch & Lomb, Rochester, NY, USA), which was the first time the phacoemulsifier was used in our hospital. At the beginning of the outbreak study, the culture results from different samples, including the irrigation fluids, were negative. Later, the aspiration liquids from the aspiration tube were positive for *S. maltophilia*. The *S. maltophilia* was Gram-negative and resulted in 2-mm purplish-green colonies on blood agar plates after 72 hours of incubation. The antibiotic susceptibility was identical to those samples collected directly from the patients. This suggested that contaminated aspiration solutions might be the source of infection. However, there should be no intraocular infection unless there was communication between the aspiration and irrigating solutions. To test this possibility, we injected an aqueous solution of dye into the aspiration tube, and found it reached the irrigation tube (Figure 4), confirming our hypothesis. The test results for two other phaco machines (Infiniti; Alcon Laboratories, Fort Worth, TX, USA and Signature; Abbott Company, Abbott Park, IL, USA) were negative. Furthermore, endophthalmitis no longer occurred after use of the Stellaris phacoemulsifier was terminated. Endophthalmitis only chronically occurred in Room 3, and after changing the location of this instrument to Room 2, the endophthalmitis only occurred in that room. Together, these results strongly suggest that the source of *S. maltophilia* was the contaminated drainage cassette.

## Discussion

The present study describes the *S. maltophilia-*induced PE of 14 patients who were infected within a period of 4 months at a single hospital because of a contaminated drainage cassette. Described in this study are the clinical characteristics, the effects of antibiotics and PPV, and the final visual acuities after treatments for *S. maltophilia* as the potential source of the infections.

In the culture-positive group, the median time from surgery to infection was 9.5 days (1–17 days). This time period was comparable with patients in a previous report [13], whose symptoms began between postoperative days 1 and 19. However, in another report [14], endophthalmitis by the same bacteria was fully developed in 26 patients within 2 days after surgery. Based upon the Endophthalmitis Vitrectomy Study (EVS), more virulent microorganisms (e.g., Gram-negative bacteria) were more likely to be diagnosed within 2 days of cataract surgery. However, in our study, as a Gram-negative bacterium, *S. maltophilia* was less virulent, and the source of *S. maltophilia* might affect the incubation time. In a previous report [14], the continuous rinsing solution, which was contaminated, could have resulted in a higher level of bacterial inoculums, resulting in quicker and more pronounced inflammation, when compared with the contaminated aspiration solution that had been diluted by irrigation solution late in our study. In addition, in our study, the latent period was significantly longer for eyes that were culture-negative compared with those that were culture-positive. This was similar to another study [17], where a shorter delayed onset was associated with a higher rate of bacterial infection.

The baseline clinical presentation of acute PE may be associated with the bacteria involved [17, 18], which may assist clinicians to better medically and surgically manage patients before microbiologic identification is made. In our study, endophthalmitis induced by *S. maltophilia* had special clinical characteristics. Pupil synechia was not seen in these patients. The liquidity of the aqueous humor was slightly influenced, and hypopyon was easy to deposit inferiorly. There was less fibrin formation and more cellular reaction in the anterior chamber. Fibrinolysin, one of the extracellular enzymes of *S. maltophilia,* could have played a role in inhibiting the process of fibrin membrane formation. In addition, retinal periphlebitis was another early clinical sign of this infection. This was perhaps due to the toxins released by *S. maltophilia*, such as lipases, hyaluronidase, protease, and elastase.

*S. maltophilia* shows intrinsic resistance to many currently used antibiotics, and therefore constitutes a special clinical challenge. Intravitreal injection of antibiotics combined with vitreous aspiration or PPV is considered standard treatment, and is usually initiated rapidly after diagnosis of PE. *S. maltophilia* showed multidrug resistance, which included amino glycosides (e.g., gentamicin, tobramycin, and amikacin), most of the β-lactams (e.g., ampicillin, ticarcillin, cefazolin, cefotaxime, cefepime, ceftriaxone, and cefuroxime), and aztreonam, imipenem, and ciprofloxacin. Based upon our study, in the process of intravitreal injection of antibiotics, the following details should be considered. First, the sensitivity of the pathogen to the antibiotics (especially the intravitreal antibiotics) was the most important factor that determined the course of the infection. Patients did not respond well after initial intravitreal ceftazidime injection when the pathogen was resistant to this drug. Improvement was observed after a subsequent intravitreal injection of levofloxacin. Second, *S. maltophilia* infections can be challenging because of contradictory findings between *in vitro* and *in vivo* antibiotic susceptibility studies. Two of the cases responded poorly to intravitreal ceftazidime, although the pathogens were reported to be sensitive to this drug. This finding is consistent with previous reports from other studies [19]. Third, the antibiotic resistance was more severe than previously reported [12]. In our study, the intravitreal-injected antibiotics such as amikacin and ceftazidime were not effective. In addition, the strain of *S. maltophilia* was also resistant to SMZ/TMP and ciprofloxacin. However, it was sensitive to levofloxacin, another fluoroquinolone drug. Fourth, in our study, levofloxacin was applied systemically as well as locally. Although patients reported in the EVS derived no demonstrable benefit from these systemic antibiotics, the study made no recommendations regarding treatment with additional antimicrobial agents (e.g., systemic fluoroquinolones) [20], so levofloxacin may ensure more predictable effects during the vulnerable period.

For the previous two patients with a visual acuity of HM, initial intravitreal injections were applied, but the infection could not be resolved. Therefore, we performed initial PPV on the remaining six patients, when their visual acuities were HM. This treatment differed from the EVS, which reported that there was no advantage to routinely performing immediate vitrectomy in patients who had better than LP visual acuity when first seen. This could be due to differences in the bacteria identified in these two studies. In the EVS, Gram-negative microorganisms were less commonly involved. Even if we performed earlier and near complete PPV, the infection was still difficult to control. The benefits of vitrectomy may be greater because of the mechanical removal of *S. maltophilia* and toxins from the eye.

In the present study, recurrence occurred in two patients (14.3%, patients 2 and 3). In another two reported outbreaks, two relapses were seen in 26 cases (7.7%), and one in six cases (16.7%) [13, 14]. Both the previous reports and our study demonstrated that *S. maltophilia* endophthalmitis may have a persistent and recurrent clinical course. For infections that were difficult to resolve, especially in patients with diabetes or high myopia with posterior capsule rupture, PPV with IOL extraction could be performed to complete the course [10, 13]. In our study, a final visual acuity of ≥20/100 was achieved by 13 of 14 patients (92.9%), compared with 80% of patients with *S. maltophilia* cultured from eye samples in a previous report [14]. Despite recurrences, visual prognosis was good, except for the diabetic patient. The diabetic patient’s final vision was HM. His serious fundus lesions led to the worst vision of all the patients. The infection aggravated the proliferative diabetic retinopathy, and total tractional retinal detachment developed. PPV with silicone oil tamponade were then performed. Furthermore, diabetic patients in the EVS had worse outcomes than nondiabetic patients [20].

*S. maltophilia* has been often associated with serious life-threatening, systemic, opportunistic infections, especially in immunosuppressed or debilitated patients [21], but it could also be found in patients with no known predisposition. Nosocomial sources of *S. maltophilia* have also been reported from the hands of health personnel, blood sampling tubes, contact lens solutions, disinfectants, nebulizers and inhalation systems, moistening water reservoirs, and ventilation tubes [19]. *S. maltophilia* is not a normal commensal on the periocular skin or conjunctiva. Its exogenous origin was reported from contaminated ophthalmic solutions (e.g., balanced salt solution, BSS) [11, 14]. In our study, we systematically eliminated these possibilities. *S. maltophilia* was found in the resterilization aspiration tube for the drainage cassette of one phacoemulsifier. This finding was consistent with another study [22], which showed that the most common microorganism cultured from the aspiration fluid of the internal vacuum control manifold (VCM) of the phaco and vitrectomy machines was *S. maltophilia*. Bacteria often colonize on plastic, and it was possible that they survived within the resterilized tubes of a contaminated biofilm. In a previous study [13], the phacoemulsifier (Alcon Legacy; Alcon Laboratories.) was possibly contaminated, but it was impossible to isolate any pathogen from either the aspiration fluid or the internal VCM [13]. In some countries, for economic reasons, the tubes that should only be used once are sometimes resterilized for each patient. These tubes are at high risk for subsequent surgeries, when the aspiration fluids can contaminate the irrigation tubes. Furthermore, perioperative anterior chamber aspirates have demonstrated a high rate of microbial contamination after cataract surgery.

Finally, a possible limitation of our study was that the pathogen involved in the negative endophthalmitis cases was perhaps not *S. maltophilia*, with the inclusion criteria including the same ocular signs, and use of the same machine during the outbreak period.

## Conclusions

*S. maltophilia* should be considered a pathogenic organism of PE. The clinical features include more cellular reaction and less fibrin formation in the anterior chamber. The resistance of *S. maltophilia* is increasing, therefore administration of an effective systemic or intravitreal antibiotic treatment and earlier PPV may contribute to a more favorable clinical course and relatively lower recurrence rate. The drainage cassette, with a connection involving aspiration fluids and irrigation tubes, should not be resterilized for reuse.

## References

[1] Hatch WV, Cernat G, Wong D, Devenyi R, Bell CM (2009). Risk factors for acute endophthalmitis after cataract surgery: a population-based study. Ophthalmology.

[2] Wykoff CC, Parrott MB, Flynn HW, Shi W, Miller D, Alfonso EC (2010). Nosocomial acute-onset postoperative endophthalmitis at a university teaching hospital (2002–2009). Am J Ophthalmol.

[3] Lauschke JL, Singh R, Wei M, Bhardwaj G, Figueira E, Montfort J (2011). Factors influencing the incidence of postoperative endophthalmitis. Am J Ophthalmol.

[4] Yao K, Zhu Y, Zhu Z, Wu J, Liu Y, Lu Y (2013). The incidence of postoperative endophthalmitis after cataract surgery in China: a multicenter investigation of 2006–2011. Br J Ophthalmol.

[5] Hanscom TA (2004). Postoperative endophthalmitis. Clin Infect Dis.

[6] Palleroni NJ, Bradbury JF (1983). Stenotrophomonas, a new bacterial genus for Xanthomonas maltophilia (Hugh 1980) Swings et al. Int J Syst Bacteriol 1993.

[7] Jucker BA, Harms H, Zehnder AJ (1996). Adhesion of the positively charged bacterium Stenotrophomonas (Xanthomonas) maltophilia 70401 to glass and Teflon. J Bacteriol.

[8] Kaiser GM, Tso PC, Morris R, McCurdy D (1997). Xanthomonas maltophilia endophthalmitis after cataract extraction. Am J Ophthalmol.

[9] Chaudhry NA, Flynn HW, Smiddy WE, Miller D (2000). Xanthomonas maltophilia endophthalmitis after cataract surgery. Arch Ophthalmol.

[10] Horio N, Horiguchi M, Murakami K, Yamamoto E, Miyake Y (2000). Stenotrophomonas maltophilia endophthalmitis after intraocular lens implantation. Graefes Arch Clin Exp Ophthalmol.

[11] Akcakaya AA, Sargin F, Erbil HH, Yazici S, Yaylali SA, Mesci C (2011). A cluster of acute-onset postoperative endophthalmitis over a 1-month period: investigation of an outbreak caused by uncommon species. Br J Ophthalmol.

[12] Chang JS, Flynn HW, Miller D, Smiddy WE (2013). Stenotrophomonas maltophilia endophthalmitis following cataract surgery: clinical and microbiological results. Clin Ophthalmol.

[13] Karakurt A, Abdik O, Sengun A, Karadag R, Saricaoglu S, Sarikatipoglu HY (2006). Stenotrophomonas maltophilia Endophthalmitis after cataract extraction. Ocul Immunol Inflamm.

[14] Horster S, Bader L, Seybold U, Eschler I, Riedel KG, Bogner JR (2009). Stenotrophomonas maltophilia induced post-cataract-surgery endophthalmitis: outbreak investigation and clinical courses of 26 patients. Infection.

[15] Kazi AA, Jermak CM, Peyman GA, Aydin E, Riazi-Esfahani M (2006). Intravitreal toxicity of levofloxacin and gatifloxacin. Ophthalmic Surg Lasers Imaging.

[16] Ferrer C, Rodriguez A, Abad JL, Fernandez J, Alio JL (2008). Bactericidal effect of intravitreal levofloxacin in an experimental model of endophthalmitis. Br J Ophthalmol.

[17] Cornut PL, Thuret G, Creuzot-Garcher C, Maurin M, Pechinot A, Bron A (2012). Relationship between baseline clinical data and microbiologic spectrum in 100 patients with acute postcataract endophthalmitis. Retina.

[18] Johnson MW, Doft BH, Kelsey SF, Barza M, Wilson LA, Barr CC (1997). The Endophthalmitis Vitrectomy Study. Relationship between clinical presentation and microbiologic spectrum. Ophthalmology.

[19] Denton M, Kerr KG (1998). Microbiological and clinical aspects of infection associated with Stenotrophomonas maltophilia. Clin Microbiol Rev.

[20] Doft BH (2008). Treatment of postcataract extraction endophthalmitis: a summary of the results from the Endophthalmitis Vitrectomy Study. Arch Ophthalmol.

[21] Chen S, Stroh EM, Wald K, Jalkh A (1992). Xanthomonas maltophilia endophthalmitis after implantation of sustained-release ganciclovir. Am J Ophthalmol.

[22] Mino de Kaspar H, Grasbon T, Kampik A (2000). Automated surgical equipment requires routine disinfection of vacuum control manifold to prevent postoperative endophthalmitis. Ophthalmology.

[CR23] The pre-publication history for this paper can be accessed here:http://www.biomedcentral.com/1471-2415/15/14/prepub

